# Orthodontic Alloy Wires and Their Hypoallergenic Alternatives: Metal Ions Release in pH 6.6 and pH 5.5 Artificial Saliva

**DOI:** 10.3390/ma17215254

**Published:** 2024-10-29

**Authors:** Zana Jusufi Osmani, Petra Tariba Knežević, Davor Vučinić, Jetmire Alimani Jakupi, Arianit A. Reka, Mustafa Can, Koray Kara, Višnja Katić

**Affiliations:** 1 Faculty of Dental Medicine, University of Rijeka, 51000 Rijeka, Croatia; zanajusufiosmani@yahoo.com (Z.J.O.); petra.tariba@uniri.hr (P.T.K.); davor.vucinic@uniri.hr (D.V.); 2 Clinical Hospital Centre Rijeka, 51000 Rijeka, Croatia; 3 Faculty of Medical Sciences, University of Tetovo, 1220 Tetovo, North Macedonia; jetmire.jalimani@unite.edu.mk; 4 Department of Chemistry, Faculty of Natural Sciences and Mathematics, University of Tetovo, 1220 Tetovo, North Macedonia; arianit.reka@unite.edu.mk; 5 Department of Engineering Sciences, Izmir Katip Celebi University, 35620 Izmir, Turkey; mustafa.can@ikc.edu.tr; 6 Graphene Application and Research Center, Izmir Katip Celebi University, 35620 Izmir, Turkey; koray.kara@ikc.edu.tr

**Keywords:** corrosion, dental alloys, orthodontics

## Abstract

Legislative framework addresses the issues of alloy corrosion, demanding the restricted use of probable carcinogenic, mutagenic, and toxic-for-human-reproduction (CMG) metals like nickel, cobalt, and chromium and demanding the development of new biomaterials. The aim of this research was to evaluate and compare the ion release of standard dental alloys and their hypoallergenic equivalents. Six types of orthodontic alloy wires (nickel–titanium (NiTi), coated NiTi, stainless steel (SS), Ni-free SS, and cobalt–chromium (CoCr) and titanium–molybdenum (TMA) were immersed into artificial saliva of pH 5.5 and 6.6. Release of metal ions was measured by inductively coupled plasma–mass spectrometry after 3, 7, 14 and 28 days. The data were analyzed using analysis of variance, and results with *p* < 0.05 were considered significant. NiTi released more Ti and Ni ions compared to the coated NiTi; SS released more iron, chromium, and nickel compared to the nickel-free SS. CoCr released cobalt in a high concentration and low amounts of chromium, nickel, and molybdenum compared to the molybdenum and titanium released by TMA. Release of metals from dental orthodontic alloys in vitro was overall lower at pH 6.6 and for the hypoallergenic equivalents when compared to standard dental alloys.

## 1. Introduction

The normal functioning of the stomatognathic system is essential for the health and psychosocial well-being of every human being, and today, there are increasing demands for aesthetic and financially accessible dental procedures to achieve health. Numerous and different artificial materials are used to restore lost, damaged, or deformed oral structures under the common name of dental materials. At one time, gold was the main dental material due to its high resistance to corrosion and suitable strength, but due to its high cost, it was pushed out of use. Today, the most commonly used are dental metal alloy materials composed of chromium (Cr), nickel (Ni), titanium (Ti), iron (Fe), molybdenum (Mo), and cobalt (Co) due to their good mechanical properties, corrosion resistance, and affordability [1,2,3]. At the same time, for each of these groups of materials, versions are offered on the market that are advertised as hypoallergenic but, at the same time, are more expensive. Many studies indicate the interaction of dental materials with oral tissue, resulting in the evolution of high-performance dental materials to meet the different demands of the oral medium [4]. Corrosion is considered the most important factor in the selection of metallic dental materials; therefore, it deserves special emphasis and must be evaluated in the oral environment, which is constantly changing [4]. Light alloys have an oxide layer that makes them resistant to further corrosion; however, these biocompatible metallic materials tend to corrode locally under changing conditions in the oral medium and degrade over time, releasing metal ions into the oral cavity [5,6] and thus contributing to general toxicity. Also, due to the frequent mechanical loads to which dental materials are exposed in the oral cavity, the protective oxide layer is constantly damaged, causing wear and permanent electrochemical corrosion [7]. Furthermore, saliva with variable PH, bacterial biofilm products, high fluoride concentrations, or gastric acid reflux as well as masticatory and orthodontic forces affect the structural and chemical stability of dental materials [8]. Previous research showed higher ex vivo results for ions release compared to the results from in vitro testing, suggesting that every in vitro result, although indicative, underestimates the clinical ion release [6].

Orthodontic fixed devices as well as partial metal skeleton prostheses and bridges usually remain exposed to degradation processes in the oral cavity for several years. The process of the degradation of dental metal alloys is the subject of in vitro research in which various parameters and their possible synergistic effects are analyzed because a complete simulation of conditions inside the oral cavity is difficult to achieve due to complex intraoral processes [9]. Almost all fixed orthodontic devices as well as the metal parts of partial dentures and bridges are made of metal alloys; among the most used materials are alloys of stainless steel (SS), cobalt–chromium (CoCr), nickel–titanium (NiTi) and titanium–molybdenum alloy (TiMo) [10]. A large number of studies that conducted allergic skin tests indicated evidence related to an allergy to dental alloys, and the metal that plays a leading role is nickel. This is of special interest for dental medicine because nickel-containing metals are widely used in orthodontics and prosthetics [11]. Nickel released from dental alloys can cause an allergic reaction in certain cases, but it is not clear whether it can also cause hypersensitivity. Schmalz and Garhammer [11] concluded that the relatively high incidence of nickel allergy should provide an incentive to replace cast Ni-based alloys with an available suitable alternative alloy. Previous research showed that some of the new products on the market do not reduce surface or general corrosion but rather increase it [12]. Also, the prevalence of oral allergies from metals that are part of various dental materials (from dental and root canal fillings to orthodontic appliances and prosthetic crowns, dentures, and implants) is increasing [13] Hypersensitivity, allergic reactions, and disease symptoms have been described, manifesting complex interactions between various components of dental materials, which came as a result of the nanoparticles intake, electromagnetic radiation, galvanic corrosion, and particular genetic individual factors [12,14]. For example, current European Council regulations ban the use of dental amalgam for treating teeth in children under 15 years old and in pregnant or breastfeeding women [15]. Furthermore, the European Union (EU) legislative framework addresses the metal alloys’ corrosion issues, demanding the restricted use of probable carcinogenic, mutagenic, and toxic-for-human-reproduction (CMG) metals like nickel, cobalt, and chromium and demanding the development of new biomaterials [16]. The findings so far confirm the need for the independent testing of new materials on the market and provide justification for using a version of the offered dental materials in clinical work the non-/justification for increased treatment costs [5,6,17,18,19].

In clinical work, for the first phase of orthodontic treatment, both standard NiTi and coated NiTi can be used due to their similar working properties. In the second phase, the standard SS and Ni-free SS wires can be alternatively used. In the third, finishing phase, either CoCr or TMA alloys can be used. The working properties for the abovementioned alloys in each phase are similar; the difference comes in the cost of the material, chemical composition, and ions released. Therefore, the aim of this research was to evaluate and compare the ion release of standard dental alloys and their hypoallergenic but more expensive equivalents.

## 2. Materials and Methods

Six types of preformed orthodontic archwire alloys were used in this study:

1. BioForce Sentalloy (Dentsply GAC, New York, NY, USA)—NiTi alloy (Ni 50.4% and Ti 49.6%);
2. High Aesthetic (Dentsply GAC, New York, NY, USA)—NiTi-coated alloy (Ni 50.4% and Ti 49.6%), with a <0.5 µm thin coating of gold and rhodium [19];
3. Remanium (Dentaurum, Ispringen, Germany)—noble steel (SS) alloy (Cr 18–20%, Ni 8–10.5%, and the rest Fe);
4. Noninium (Dentaurum, Ispringen, Germany)—noble steel (SS Ni-free) alloy (Mn 16–20%, Cr 16–20%, Mo 1.8–2.5%, and the rest Fe);
5. Elgiloy (Dentaurum, Ispringen, Germany)—alloy CoCr (Ni 14–16%, Cr 19–21%, Mo 6–8%, and Co 38–42%);
6. Rematitan Special (Dentaurum, Ispringen, Germany)—TiMo alloy (TMA) (Mo 11.5% and Ti 78%).

Artificial saliva was prepared from 1.5 g/L KCl, 1.5 g/L NaHCO_3_, 0.5 g/L 0.5 g/L KSCN, and 0.9 g/L lactic acid. The pH values were adjusted, with lactic acid imitating a neutral oral environment (pH 6.6) and the oral environment in the case of poor oral hygiene (pH 5.5) [17].

Samples of the alloys of dental materials were prepared in laboratory conditions at the University of Tetovo. Alloy samples were immersed in 10 mL of sterile artificial saliva (1 cm of sample alloy/1 mL of artificial saliva) for a period of 3, 7, 14, and 28 days at 37 °C and with moderate shaking on a mechanical shaker (100 rpm). Samples containing saliva without alloys served as the negative control.

All measurements were taken on five samples of each type of examined dental material in combination with different pH of artificial saliva. Sample size was calculated according to the power analysis of previous research, with the power of effect set at 0.8, alpha level at 0.05, and a presumed effect size of 0.7 [20]. Every sample was measured once at the end of every immersion time; the wire sample was transferred into fresh artificial saliva sample until the total incubation time.

Ion release was recorded using inductively coupled plasma–mass spectrometry (ICP-MS) (Agilent Technologies 7800, Santa Clara, CA, USA), and all specimens were acidified with one drop of concentrated HNO_4_ before injection into the spectrometer. The release of metals was expressed as µg/cm^2^ to enable better evaluation of the aspect of biocompatibility by translating experimental results into clinical conditions, as described by Arndt et al. [21]. Data presented in this way enable the calculation of the average daily release for, e.g., two wires (for upper and lower dental arch, total length 28 cm), as expected in clinical conditions, for persons with good (pH 6.6) or bad oral hygiene (pH 5.5) or any other clinical situation regarding the type and dimensions of wires.

Statistical analysis for all obtained quantitative data was performed using statistical software R 4.3.1. (R Core Team, 2023) [22]. The Shapiro–Wilk test was used as the basic test of normality of distributions. In cases of deviations from the normal distribution, the skewness and kurtosis of the distributions were calculated, and 3 for flatness and 7 for curvature were taken as reference values according to Kline’s recommendations [23]. Data analyses were performed using analysis of variance, which allows testing for differences. Pairwise comparisons were calculated using Benjamini–Hochberg correction for the *p*-values. Results with *p* < 0.05 were considered significant.

## 3. Results

### 3.1. Nickel Release

**Figure 1:**
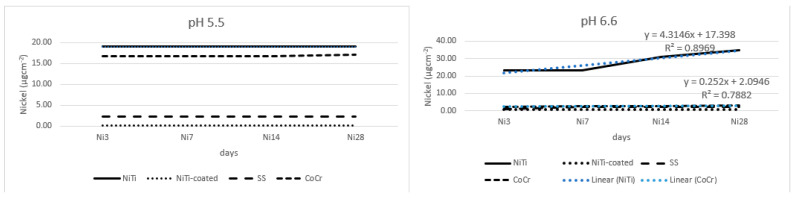
Nickel release from the tested alloys.

Results for the nickel release are presented in Figure 1, with equations for linear trendline and *R*^2^-value, where possible. At lower pH, NiTi and CoCr released mostly nickel, while at pH 6.6, again, NiTi released mostly nickel, while other alloys showed minimum release. At pH 5.5, release was highest at the first time point, and later, it was minimal or below detection. At pH 6.6, release was initially high and again increased after 14 days of immersion. There was more nickel released at pH 6.6, probably due to the crevice corrosion caused by surface irregularities. Where there was no variability between time points, further analyses could not be performed.

### 3.2. Titanium Release

**Figure 2:**
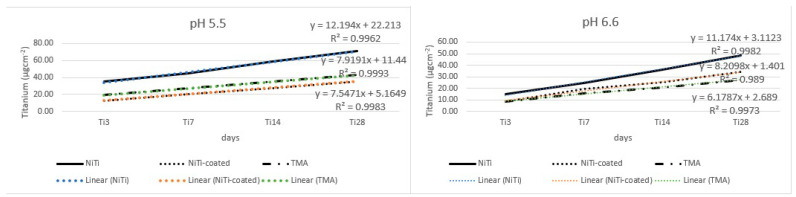
Titanium release from the tested alloys.

**Figure 3:**
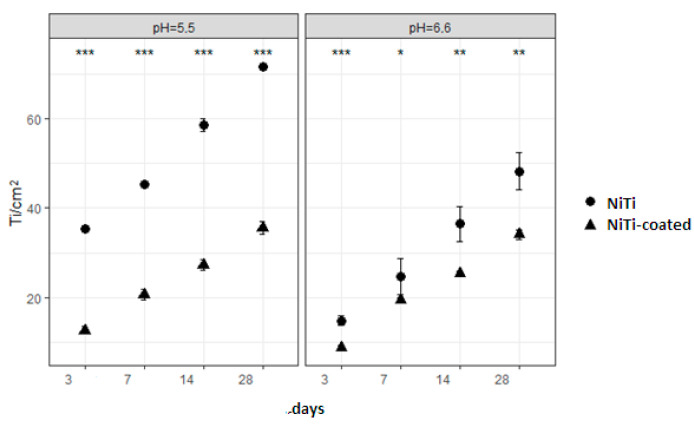
Comparison of titanium release from NiTi and NiTi-coated alloys for every time point in pH 5.5 (**left**) and pH 6.6 (**right**) shows significant differences (*** *p* < 0.001; ** *p* < 0.01; * *p* < 0.05).

Figure 2 shows release of titanium. The highest release was noted for NiTi in both pH settings; TMA had second highest release in pH 5.5, while in pH 6.6, it was the NiTi-coated alloy. Titanium showed an increase in release over time in both pH settings. Analysis of the main effects for NiTi and NiTi-coated alloys, regarding release of titanium, showed significant differences for all time points in both pH settings (Table 1 and Figure 3). The overall release of titanium was higher than the release of nickel, probably due to the greater presence of TiO_2_ on the surface, which needed to be resolved prior to the dissolution of nickel from subsurface.

### 3.3. Iron Release

**Figure 4:**
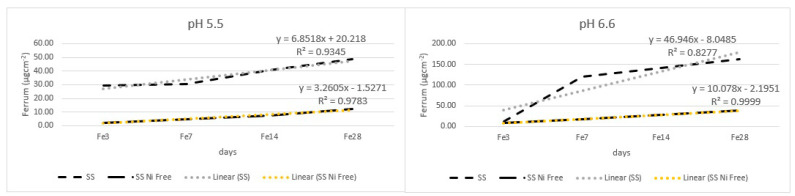
Iron (ferrum) released from dental alloys.

**Figure 5:**
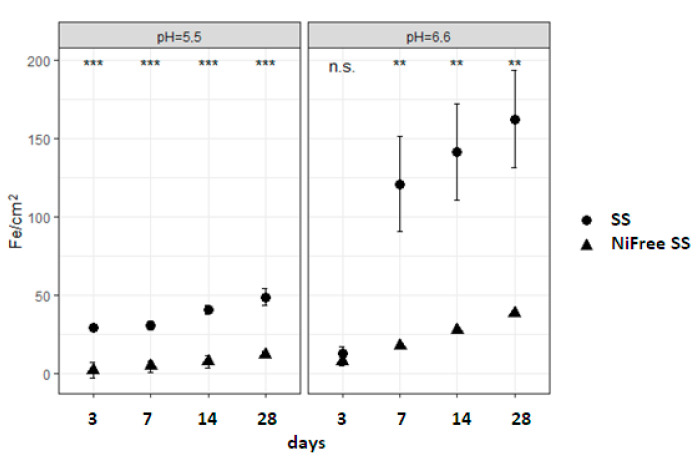
Comparison of iron released from SS and SS Ni-free alloys for every time point in pH 5.5 (**left**) and pH 6.6 (**right**) shows significant differences (n.s., not significant; *** *p* < 0.001; ** *p* < 0.01).

In Figure 4, the graphs show the iron release under both pH values. The SS alloy had a higher release of iron under both pH values when compared to the nickel-free SS alloy but only after 7 days of immersion, probably due to surface irregularities caused by manipulation. The amounts released under higher pH were three times higher than those released under lower pH. Table 2 and Figure 5 show that all main effects and all interactions are statistically significant, although they showed lower but still large effects when compared to other metals.

### 3.4. Chromium Release

**Figure 6:**
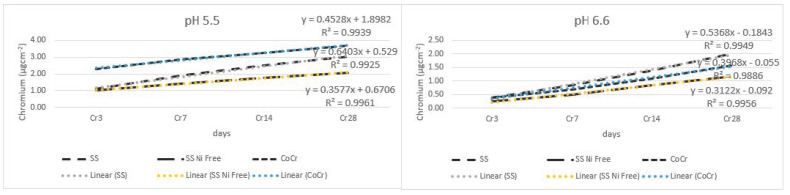
Release of chromium from wire alloys.

Figure 6 shows results of the release of chromium in all tested alloys. In lower pH, most chromium was released in the following order: CoCr < SS < SS Ni-free alloy.

**Figure 7:**
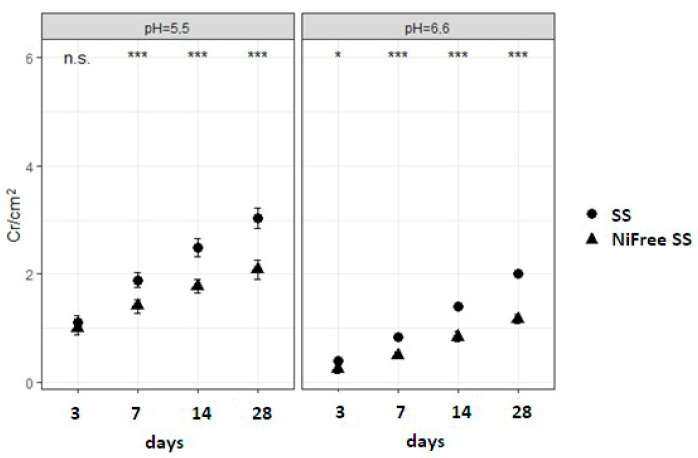
Comparison of chromium released from SS and SS Ni-free alloys for every time point in pH 5.5 (**left**) and pH 6.6 (**right**) shows significant differences (n.s., not significant; *** *p* < 0.001; * *p* < 0.05).

Table 3 and Figure 7 show that all the main effects for the SS and SS Ni-free alloys were significant, with large effects due to alloy type, pH of saliva, and time of immersion; out of all the interactions, only the interaction between alloy type and pH value was insignificant.

### 3.5. Cobalt Release

**Figure 8:**
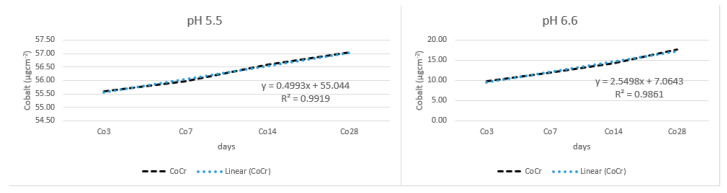
Release of cobalt from alloy wire.

Figure 8 shows release of cobalt from the CoCr alloy. At lower pH, the release of cobalt was much higher, approximately four times higher.

### 3.6. Manganese Release

**Figure 9:**
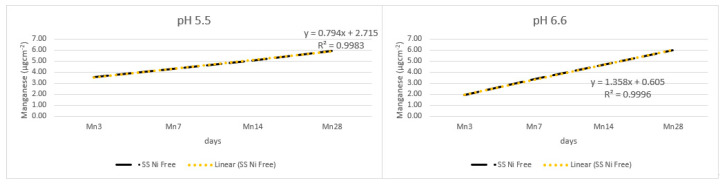
Release of manganese from the alloy wires.

Figure 9 shows the release of manganese from the tested alloys; the recorded release was low overall, and the cumulative amount was similar in both pH settings.

### 3.7. Molybdenum Release

**Figure 10:**
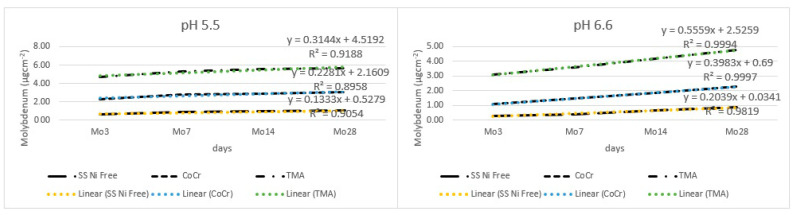
Release of molybdenum from alloy wires.

Figure 10 shows release of molybdenum from the tested alloys. Under the lower pH, the release took the following order: TMA > CoCr > SS Ni-free alloy. The overall release was low.

**Figure 11:**
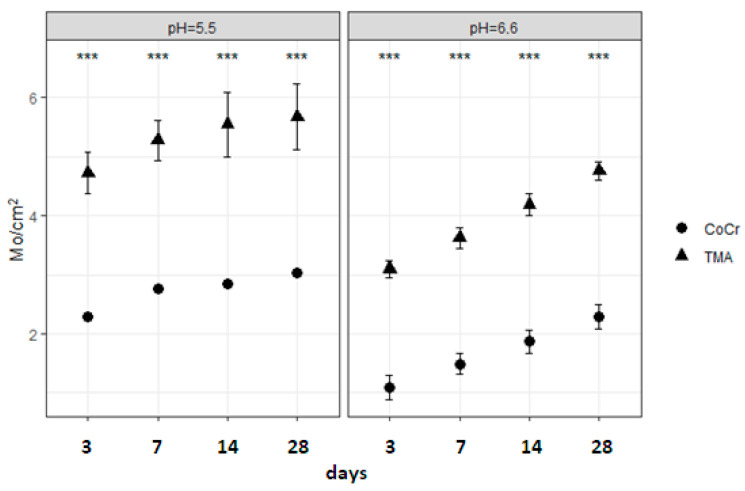
Comparison of molybdenum released from CoCr and TMA alloys for every time point in pH 5.5 (**left**) and pH 6.6 (**right**) shows significant differences (*** *p* < 0.001) at every time point.

The results from Table 4 and Figure 11 show that all main effects were statistically significant, with large effects due to alloy, pH, and time. Out of all the interactions, the interaction between alloy type and pH value and the interaction between type of alloy, pH value, and time were not significant.

## 4. Discussion

The ion release from the tested dental alloys was lower from the proposed hypoallergenic variants when compared to the standard materials. The greatest amount of nickel was released from the uncoated NiTi wires and the stainless-steel wires, while their counterparts had minimal or no nickel release. Also, after initial low release at pH 6.6, the release of Ni from uncoated NiTi increased substantially, probably due to manipulation with metal tweezers in between the two time points [24,25] and the emergence of the subsurface material after the dissolution of the surface protective layer. A similar result was observed for the iron released from the SS, also probably enhanced by manipulation and the formation of the unstable ferrous compound, which dissolve quickly in near-neutral conditions [25,26]. Previous research by Laird et al. also found that hypoallergenic alloys release less metallic ions when compared to the standard alloys [27] and that the type and uniformity of coating influence ion release [17,18]. Research by Edward Cho et al. showed that even thickened rhodium coating on NiTi wires leaches nickel, but the overall amount is low and almost undetectable in subcutaneous tissue [28]. The levels of released nickel and titanium in our study are comparable to those of Chikhale et al. [7], who studied NiTi and TMA wires. Previous research showed that Ni is the most common cause of allergy, and it also acts as a catalyst, increasing risk of sensitization and allergy to cobalt [29]. As our results showed, the CoCr alloy released a high amount of the cobalt (among the highest values of all measured metals) along with a high amount of nickel, and as mentioned, the combined effect of both ions poses a risk to human health [29]. The interactions and even catalytic effects provide another reason to keep the overall leaching from dental alloys as low as possible.

The titanium release was also significantly greater in the classic NiTi when compared to the coated NiTi, contrary to previous research on coated NiTi wires [18], suggesting improvement in the coating quality. Recent research has shown more and more cases of adverse reactions in dental patients, caused by the various combinations of galvanic coupling and influenced by a number of extrinsic (various radiation sources) and intrinsic hereditary factors [12].Research has shown that accumulation of titanium (and iron) increased the sensitivity of gingival epithelial cells to microorganisms in the oral cavity and involved the lymphocytes, macrophages, monocytes, and osteoclasts, deteriorating both soft and hard tissue in peri-implantitis cases [30,31]. Therefore, the release of iron should be also kept to a minimum to decrease inflammation and the susceptibility to infections of oral mucosa and alveolar bone. In our research, the release of iron was significantly lower in Ni-free SS when compared to the classic SS alloy. It is noteworthy that a higher release of iron was also recorded after some time of immersion in higher pH, probably due to manipulation scratches and the formation of unstable ferrous compounds [25,26].

There is a continuous trend that tends to keep the uptake of the metals, metallic ions, and nanoparticles as low as possible based on the recommendations and legislation of the EU, International Agency for Research on Cancer (IARC) of the World Health Organization (WHO), and the U.S. National Toxicology Program (NTP) and Food and Drug Administration (FDA) [16,32,33,34]. With new labelling and consumer warnings becoming mandatory, for alloys containing a CMR substance like nickel, cobalt, or chromium, it seems advisable to use alloys with lower quantities of the implicated materials or without them. Our research showed that four times higher concentrations of cobalt were released from the CoCr alloy in comparison to the counterpart wire made mainly from titanium and molybdenum, which makes it less recommendable for use. Also, CoCr released all of the above-mentioned Ni, Co, and Cr and additionally Mo, which makes it the least desirable option among the available alloys. The release of titanium from the TMA wire was comparable to results found in previous research [7].

Manganese is an essential element involved in several physiological functions of the human body, but ongoing Mn accumulation leads to the increased formation of reactive oxygen species (ROS), which contributes to mitochondrial dysfunction, DNA fragmentation, and apoptosis [35]. Overall, the released manganese levels were low but still higher for the standard SS alloy when compared to the hypoallergenic counterpart, with the time of immersion as the most significant factor.

The recommended dietary allowances (RDAs) describe the daily dietary levels of uptake of molybdenum for different age groups, suggesting that children of different age groups are rather sensitive to certain uptake levels, although the evidence on its influence on human health is limited [36]. Data on the immunotoxicity of molybdenum are also limited, with approximately 3% of patients with stainless steel stents also having delayed contact hypersensitivity to molybdenum. Further studies on influence of molybdenum on human health are necessary [37]. Molybdenum is added to hypoallergenic Ni-free SS to improve its corrosion resistance [38], and the release of Mo from this alloy was the least among all observed alloys. Also, the release of iron from Ni-free SS is much lower than from the standard SS alloy; therefore, the potential harmful effect from the combination of both is low.

Mineral sources and elements such as chromium, iron, and manganese, among others, are essential for humans because they are involved in a variety of metabolic functions, enzyme activities, receptor sites, hormonal function, and protein transport at specific concentrations; they can be regulated to some extent, but excessive overload can result in a number of unfavorable cellular responses, from the production of ROS to DNA and protein damage, cell apoptosis, and carcinogenesis [39,40,41,42].

The overall intake of the various elements should be kept as low as possible, as various interactions between metals in human body are still to be investigated in detail. Our research showed that coated NiTi wires should be recommended over standard NiTi, Ni-free stainless steel should be recommended over standard stainless steel, and the titanium–molybdenum alloy should be the preferred choice over cobalt–chromium alloys. In this way, the overall intake of various metals would be kept to a minimum. Also, as legislative bodies have made distinctions between children’s and adults’ recommended daily uptakes, it is even more important to keep the overall daily intake as low as possible in children.

The limitation to our research is that the explored release of ions involved in vitro conditions, and the research shows that the actual ion release could be even higher in vivo [6,24,25], posing more threat to human health, especially under the reduced antioxidant capacity of immunocompromised persons or with the added risks from other sources of metal ion release (either dental rehabilitation, artificial hips, stents, etc.) [26,38,42].

## 5. Conclusions

The release of metals from dental orthodontic alloys in vitro is overall lower for the hypoallergenic equivalents when compared to the standard dental alloys. The lower pH of artificial saliva mainly caused higher initial and overall metal ions release.

NiTi released more of titanium and nickel metal ions when compared to the coated NiTi.

Stainless steel released more iron, chromium, and nickel when compared to the nickel-free SS.

CoCr released cobalt in a high concentration and low amounts of chromium, nickel, and molybdenum compared to the molybdenum and titanium released by TMA.

## Figures and Tables

**Table 1:** Analysis of the main effects using analysis of variance for repeated measurements with testing of the effects of pH, type of alloy, and time (mixed ANOVA, for the titanium released from NiTi and NiTi-coated alloys.

Effect	DFn ^1^	DFd ^2^	F ^3^	*p* ^4^	ges ^5^
Alloy	1.0	16.00	703.27	0.000	0.967
pH	1.0	16.00	277.97	0.000	0.921
Time	1.5	23.93	1946.00	0.000	0.975
Alloy/pH	1.0	16.00	188.09	0.000	0.888
Alloy/time	1.5	23.93	79.59	0.000	0.619
pH/time	1.5	23.93	1.31	0.280	0.026
Alloy/pH/time	1.5	23.93	3.79	0.048	0.072

^1^ Degrees of freedom for the number of levels; ^2^ degrees of freedom for the number of measurements; ^3^ F ratio; ^4^ *p*-value; ^5^ generalized eta square.

**Table 2:** Analysis of the main effects using analysis of variance for repeated measurements with testing of the effects of pH, type of alloy, and time (mixed ANOVA) for the iron released from SS and SS Ni-free alloys.

Effect	DFn ^1^	DFd ^2^	F ^3^	*p* ^4^	ges ^5^
Alloy	1.00	16.00	116.44	0.000	0.852
pH	1.00	16.00	66.34	0.000	0.767
Time	1.04	16.72	198.19	0.000	0.720
Alloy/pH	1.00	16.00	26.27	0.000	0.566
Alloy/time	1.04	16.72	83.05	0.000	0.518
pH/time	1.04	16.72	111.83	0.000	0.592
Alloy/pH/time	1.04	16.72	66.41	0.000	0.462

^1^ Degrees of freedom for the number of levels; ^2^ degrees of freedom for the number of measurements; ^3^ F ratio; ^4^ *p*-value; ^5^ generalized eta square.

**Table 3:** Analysis of the main effects using analysis of variance for repeated measurements with testing of the effects of pH, type of alloy, and time (mixed ANOVA) for the chromium released from SS and SS Ni-free alloys.

Effect	DFn ^1^	DFd ^2^	F ^3^	*p* ^4^	ges ^5^
Alloy	1.00	16.00	105.94	0.000	0.864
pH	1.00	16.00	338.63	0.000	0.953
Time	1.78	28.46	10,382.57	0.000	0.962
Alloy/pH	1.00	16.00	0.91	0.355	0.052
Alloy/time	1.78	28.46	783.81	0.000	0.657
pH/time	1.78	28.46	123.36	0.000	0.232
Alloy/pH/time	1.78	28.46	18.75	0.000	0.044

^1^ Degrees of freedom for the number of levels; ^2^ degrees of freedom for the number of measurements; ^3^ F ratio; ^4^ *p*-value; ^5^ generalized eta square.

**Table 4:** Analysis of the main effects using analysis of variance for repeated measurements with testing of the effects of pH, type of alloy, and time (mixed ANOVA) for the molybdenum released from CoCr and TMA alloys.

Effect	DFn ^1^	DFd ^2^	F ^3^	*p* ^4^	ges ^5^
Alloy	1.00	16.0	448.72	0.000	0.963
pH	1.00	16.0	115.28	0.000	0.869
Time	1.07	17.2	656.49	0.000	0.760
Alloy/pH	1.00	16.0	2.30	0.149	0.117
Alloy/time	1.07	17.2	18.04	0.000	0.080
pH/time	1.07	17.2	58.63	0.000	0.221
Alloy/pH/time	1.07	17.2	2.11	0.164	0.010

^1^ Degrees of freedom for the number of levels; ^2^ degrees of freedom for the number of measurements; ^3^ F ratio; ^4^ *p*-value; ^5^ generalized eta square.

## Data Availability

The datasets presented in this article are not readily available because the data are part of an ongoing study. Requests to access the datasets should be directed to visnja.katic@uniri.hr.

## References

[1] Upadhyay D., Panchal M.A., Dubey R.S., Srivastava V.K. (2006). Corrosion of alloys used in dentistry: A review. Mater. Sci. Eng. A.

[2] Westphalen G.H., Menezes L.M., Prá D., Garcia G.G., Schmitt V.M., Henriques J.A., Medina-Silva R. (2008). In vivo determination of genotoxicity induced by metals from orthodontic appliances using micronucleus and comet assays. Genet. Mol. Res..

[3] Wichelhaus A., Geserick M., Hibst R., Sander F.G. (2005). The effect of surface treatment and clinical use on friction in NiTi orthodontic wires. Dent. Mater..

[4] Quadras D.D., Nayak U.S.K., Kumari N.S., Priyadarshini H.R., Gowda S., Fernandes B. (2019). In vivo study on the release of nickel, chromium, and zinc in saliva and serum from patients treated with fixed orthodontic appliances. Dent. Res. J..

[5] Rongo R., Valletta R., Bucci R., Rivieccio V., Galeotti A., Michelotti A., D’Antò V. (2016). In vitro biocompatibility of nickel-titanium esthetic orthodontic archwires. Angle Orthod..

[6] Kovac V., Poljsak B., Bergant M., Scancar J., Mezeg U., Primozic J. (2022). Differences in Metal Ions Released from Orthodontic Appliances in an In Vitro and In Vivo Setting. Coatings.

[7] Chikhale R., Akhare P., Umre U., Jawlekar R., Kalokhe S., Badole N., Beri A. (2024). In Vitro Comparison to Evaluate Metal Ion Release: Nickel-Titanium vs. Titanium-Molybdenum Orthodontic Archwires. Cureus.

[8] Močnik P., Kosec T., Kovač J., Bizjak M. (2017). The effect of pH, fluoride and tribocorrosion on the surface properties of dental archwires. Mater. Sci. Eng. C.

[9] Noguti J., de Oliveira F., Peres R.C., Renno A.C.M., Ribeiro D.A. (2012). The role of fluoride on the process of Ti corrosion in oral cavity. Biometals.

[10] Messer R., Wataha J., Buschow J.K.H., Cahn R.W., Flemings M.C., Ilschner B., Kramer E.J., Mahajan S., Veyssière P. (2002). Dental Materials: Biocompatibility. Encyclopedia of Materials: Science and Technology.

[11] Schmalz G., Garhammer P. (2002). Biological interactions of dental cast alloys with oral tissues. Dent. Mater..

[12] Tibau A.V., Grube B.D., Velez B.J., Vega V.M., Mutter J. (2019). Titanium exposure and human health. Oral Sci. Int..

[13] Evrard L., Waroquier D., Parent D. (2010). Allergies to dental metals. Ti: A new allergen. Rev. Med. Brux..

[14] Lee J.J., Song K.Y., Ahn S.G., Choi J.Y., Seo J.M., Park J.M. (2015). Evaluation of effect of galvanic corrosion between nickel-chromium metal and Ti on ion release and cell toxicity. J. Adv. Prosthodont..

[15] Regulation of the European Parliament and of the Council. Amending Regulation (EU) 2017/852 on Mercury as Regards Dental Amalgam and Other Mercury-Added Products Subject to Export, Import and Manufacturing Restrictions. https://data.consilium.europa.eu/doc/document/PE-53-2024-INIT/en/pdf.

[16] Vaicelyte A., Janssen C., Le Borgne M., Grosgogeat B. (2020). Cobalt–Chromium Dental Alloys: Metal Exposures, Toxicological Risks, CMR Classification, and EU Regulatory Framework. Crystals.

[17] Katić V., Curković H.O., Semenski D., Baršić G., Marušić K., Spalj S. (2014). Influence of surface layer on mechanical and corrosion properties of nickel-titanium orthodontic wires. Angle Orthod..

[18] Jusufi Osmani Z., Poljšak B., Zelenika S., Kamenar E., Marković K., Perčić M., Katić V. (2022). Ion Release and Surface Changes of Nickel–Titanium Archwires Induced by Changes in the pH Value of the Saliva—Significance for Human Health Risk Assessment. Materials.

[19] Iijima M., Muguruma T., Brantley W., Choe H.C., Nakagaki S., Alapati S.B., Mizoguchi I. (2012). Effect of coating on properties of esthetic orthodontic nickel-titanium wires. Angle Orthod..

[20] Jamilian A., Moghaddas O., Toopchi S., Perillo L. (2014). Comparison of nickel and chromium ions released from stainless steel and NiTi wires after immersion in Oral B^®^, Orthokin^®^ and artificial saliva. J. Contemp. Dent. Pract..

[21] Arndt M., Brück A., Scully T., Jäger A., Bourauel C. (2005). Nickel ion release from orthodontic Ni-Ti wires under simulation of realistic in-situ conditions. J. Mater. Sci..

[22] R Core Team (2013). R: A Language and Environment for Statistical Computing.

[23] Kline R.B. (2016). Principles and Practice of Structural Equation Modelling.

[24] Huang H.H., Chiu Y.H., Lee T.H., Wu S.C., Yang H.W., Su K.H., Hsu C.C. (2003). Ion release from NiTi orthodontic wires in artificial saliva with various acidities. Biomaterials.

[25] Daems J., Celis J.P., Willems G. (2009). Morphological characterization of as-received and in vivo orthodontic stainless steel archwires. Eur. J. Orthod..

[26] Fróis A., Santos A.C., Louro C.S. (2023). Corrosion of Fixed Orthodontic Appliances: Causes, Concerns, and Mitigation Strategies. Metals.

[27] Laird C., Xu X., Yu Q., Armbruster P., Ballard R. (2021). Nickel and chromium ion release from coated and uncoated orthodontic archwires under different pH levels and exposure times. J. Oral Biosci..

[28] Cho E., Kanno Z., Yonemitsu I., Kiyokawa H., Ohira N., Ono T., Uo M. (2022). Effect of rhodium plating on the ion dissolution from nickel-titanium and pure nickel wires. Asian Pac. J. Dent..

[29] Bonefeld C.M., Nielsen M.M., Vennegaard M.T., Johansen J.D., Geisler C., Thyssen J.P. (2015). Nickel acts as an adjuvant during cobalt sensitization. Exp. Dermatol..

[30] Fretwurst T., Buzanich G., Nahles S., Woelber J.P., Riesemeier H., Nelson K. (2016). Metal elements in tissue with dental peri-implantitis: A pilot study. Clin. Oral Implants Res..

[31] Wachi T., Shuto T., Shinohara Y., Matono Y., Makihira S. (2015). Release of Ti ions from an implant surface and their effect on cytokine production related to alveolar bone resorption. Toxicology.

[32] IARC Monographs on the Evaluation of Carcinogenic Risks to Humans. Chromium, Nickel and Welding. Volume 49. https://publications.iarc.fr/67.

[33] National Toxicology Program. https://ntp.niehs.nih.gov/pubhealth/roc/index-1.html.

[34] Biological Responses to Metal Implants. FDA Report Published on September 2019. https://www.raps.org/news-and-articles/news-articles/2021/8/an-overview-of-recent-fda-activity-on-materials-in.

[35] Harischandra D.S., Ghaisas S., Zenitsky G., Jin H., Kanthasamy A., Anantharam V., Kanthasamy A.G. (2019). Manganese-Induced Neurotoxicity: New Insights Into the Triad of Protein Misfolding, Mitochondrial Impairment, and Neuroinflammation. Front. Neurosci..

[36] Agency for Toxic Substances and Disease Registry, Division of Toxicology and Human Health Sciences, Environmental Toxicology Branch, U.S. Department of Health and Human Services Toxicological Profile for Molybdenum: Draft for Public Comment. External Link Disclaimer April 2017. https://www.atsdr.cdc.gov/toxprofiles/tp212.pdf.

[37] Köster R., Vieluf D., Kiehn M., Sommerauer M., Kähler J., Baldus S., Meinertz T., Hamm C.W. (2000). Nickel and molybdenum contact allergies in patients with coronary in-stent restenosis. Lancet.

[38] Eliaz N. (2019). Corrosion of Metallic Biomaterials: A Review. Materials.

[39] Jaishankar M., Tseten T., Anbalagan N., Mathew B.B., Beeregowda K.N. (2014). Toxicity, mechanism and health effects of some heavy metals. Interdiscip. Toxicol..

[40] Kim K.T., Eo M.Y., Nguyen T.T.H., Kim S.M. (2019). General review of titanium toxicity. Int. J. Implant. Dent..

[41] Grosgogeat B., Vaicelyte A., Gauthier R., Janssen C., Le Borgne M. (2022). Toxicological Risks of the Cobalt–Chromium Alloys in Dentistry: A Systematic Review. Materials.

[42] Primožič J., Poljšak B., Jamnik P., Kovač V., Čanadi Jurešić G., Spalj S. (2021). Risk Assessment of Oxidative Stress Induced by Metal Ions Released from Fixed Orthodontic Appliances during Treatment and Indications for Supportive Antioxidant Therapy: A Narrative Review. Antioxidants.

